# Supplementary material for: MetaQTL: a package of new computational methods for the meta-analysis of QTL mapping experiments
Source: BMC Bioinformatics. 2007 Feb 8;8:49. doi: 10.1186/1471-2105-8-49 (PMC1808479; doi:10.1186/1471-2105-8-49)
Supplement: Additional File 7 — MetaQTL Package : jar file and tutorial. This Zip archive contains both the MetaQTL JAR file and the files of the tutorial. [file 1471-2105-8-49-S7.zip › org.inra.metaqtl/doc/org/metaqtl/EMCriteria.html]

EMCriteria


|  |  |  |
| --- | --- | --- |
| **Overview** \| **Package** \| **Class** \| **Use** \| **Tree** \| **Deprecated** \| **Index** \| **Help** | |  |
| **PREV CLASS**   **NEXT CLASS** | **FRAMES**    **NO FRAMES**     **All Classes** |  |
| SUMMARY: NESTED \| FIELD \| CONSTR \| METHOD | DETAIL: FIELD \| CONSTR \| METHOD |  |


---


## org.metaqtl Class EMCriteria

```
java.lang.Object
  org.metaqtl.EMCriteria
```

**All Implemented Interfaces:**: java.io.Serializable

---

``` public class EMCriteria extends java.lang.Object implements java.io.Serializable ```

**Author:**
:   jveyrier

**See Also:**: Serialized Form

---

| **Field Summary** | |
| --- | --- |
| `double` | `aic` |
| `static int` | `AIC` |
| `static java.lang.String` | `AIC_NAME` |
| `double` | `aic3` |
| `static int` | `AIC3` |
| `static java.lang.String` | `AIC3_NAME` |
| `double` | `aicc` |
| `static int` | `AICc` |
| `static java.lang.String` | `AICc_NAME` |
| `double` | `awe` |
| `static int` | `AWE` |
| `static java.lang.String` | `AWE_NAME` |
| `double` | `bic` |
| `static int` | `BIC` |
| `static java.lang.String` | `BIC_NAME` |
| `double` | `icomp` |
| `static int` | `ICOMP` |
| `static java.lang.String` | `ICOMP_NAME` |
| `double` | `mir` |


**Constructor Summary**

`EMCriteria()`


| **Method Summary** | |
| --- | --- |
| `static EMResult` | `getBestResult(EMResult[] clusterings, int criterion)`             For the given criterion `criterion` this methods looks into the array of `EMResult` and find the model which is optimal. |
| `static EMResult` | `getBestResult(EMResult[] clusterings, int n, int criterion)` |
| `static EMResult` | `getBestResult(EMResult[] clusterings, int n, java.lang.String criterion)` |
| `static EMResult` | `getBestResult(EMResult[] clusterings, java.lang.String criterion)` |
| `static java.util.Iterator` | `getCriteria()`             Returns an iterator on the criteria |
| `double` | `getCriterion(int criterion)`             Get the criterion value according to its integer code. |
| `double` | `getCriterion(java.lang.String criterion)` |
| `static int` | `getCriterionIdx(java.lang.String name)`             Returns the integer code for the criterion which matches the given name. |

**Methods inherited from class java.lang.Object**

`clone, equals, finalize, getClass, hashCode, notify, notifyAll, toString, wait, wait, wait`

**Field Detail**

### AIC

```
public static final int AIC
```

**See Also:**: Constant Field Values

---


### AIC3

```
public static final int AIC3
```

**See Also:**: Constant Field Values

---


### ICOMP

```
public static final int ICOMP
```

**See Also:**: Constant Field Values

---


### BIC

```
public static final int BIC
```

**See Also:**: Constant Field Values

---


### AWE

```
public static final int AWE
```

**See Also:**: Constant Field Values

---


### AICc

```
public static final int AICc
```

**See Also:**: Constant Field Values

---


### AIC\_NAME

```
public static final java.lang.String AIC_NAME
```

**See Also:**: Constant Field Values

---


### AICc\_NAME

```
public static final java.lang.String AICc_NAME
```

**See Also:**: Constant Field Values

---


### AIC3\_NAME

```
public static final java.lang.String AIC3_NAME
```

**See Also:**: Constant Field Values

---


### ICOMP\_NAME

```
public static final java.lang.String ICOMP_NAME
```

**See Also:**: Constant Field Values

---


### BIC\_NAME

```
public static final java.lang.String BIC_NAME
```

**See Also:**: Constant Field Values

---


### AWE\_NAME

```
public static final java.lang.String AWE_NAME
```

**See Also:**: Constant Field Values

---


### aic

```
public double aic
```

---


### aicc

```
public double aicc
```

---


### aic3

```
public double aic3
```

---


### bic

```
public double bic
```

---


### mir

```
public double mir
```

---


### icomp

```
public double icomp
```

---


### awe

```
public double awe
```


**Constructor Detail**

### EMCriteria

```
public EMCriteria()
```


**Method Detail**

### getCriterion

```
public double getCriterion(int criterion)
```

:   Get the criterion value according to its integer
    code.

    :   **Parameters:**: `criterion` - **Returns:**: the value of the criterion.

---


### getBestResult

```
public static EMResult getBestResult(EMResult[] clusterings,
                                     int criterion)
```

:   For the given criterion `criterion` this methods
    looks into the array of `EMResult` and find the model
    which is optimal.

    :   **Parameters:**: `clusterings` -: `criterion` - **Returns:**: the result which is optimal according to the criterion.

---


### getBestResult

```
public static EMResult getBestResult(EMResult[] clusterings,
                                     int n,
                                     int criterion)
```

---


### getBestResult

```
public static EMResult getBestResult(EMResult[] clusterings,
                                     java.lang.String criterion)
```

:   **Parameters:**: `clusterings` -: `criterion` - **Returns:**

---


### getBestResult

```
public static EMResult getBestResult(EMResult[] clusterings,
                                     int n,
                                     java.lang.String criterion)
```

:   **Parameters:**: `clusterings` -: `criterion` - **Returns:**

---


### getCriteria

```
public static java.util.Iterator getCriteria()
```

:   Returns an iterator on the criteria

    :   **Returns:**

---


### getCriterionIdx

```
public static int getCriterionIdx(java.lang.String name)
```

:   Returns the integer code for the criterion which matches
    the given name.

    :   **Parameters:**: `name` - **Returns:**

---


### getCriterion

```
public double getCriterion(java.lang.String criterion)
```

:   **Parameters:**: `criterion` - **Returns:**


---


|  |  |  |
| --- | --- | --- |
| **Overview** \| **Package** \| **Class** \| **Use** \| **Tree** \| **Deprecated** \| **Index** \| **Help** | |  |
| **PREV CLASS**   **NEXT CLASS** | **FRAMES**    **NO FRAMES**     **All Classes** |  |
| SUMMARY: NESTED \| FIELD \| CONSTR \| METHOD | DETAIL: FIELD \| CONSTR \| METHOD |  |


---
